# The prevalence of metabolic disorders in various phenotypes of polycystic ovary syndrome: a community based study in Southwest of Iran

**DOI:** 10.1186/1477-7827-12-89

**Published:** 2014-09-16

**Authors:** Fahimeh Ramezani Tehrani, Homeira Rashidi, Mahnaz Bahri Khomami, Maryam Tohidi, Fereidoun Azizi

**Affiliations:** Reproductive Endocrinology Research Center, Research Institute for Endocrine Sciences, Shahid Beheshti University of Medical Sciences, Tehran, Iran; Diabetes Research Center, Ahvaz Jundishapur University of Medical Sciences, Ahvaz, Iran; Prevention of Metabolic Disorders Research Center, Research Institute for Endocrine Sciences, Shahid Beheshti University of Medical Sciences, Tehran, Iran; Endocrine Research Center, Research Institute for Endocrine Sciences, Shahid Beheshti University of Medical Sciences, Tehran, Iran

**Keywords:** PCOS, Metabolic disorders, Metabolic syndrome, Phenotype

## Abstract

**Background:**

Polycystic ovary syndrome (PCOS) is a common endocrinopathy, associated with metabolic abnormalities. Metabolic features of various phenotypes of this syndrome are still debatable. The aim of present study hence was to evaluate the metabolic and hormonal features of PCOS phenotypes in comparison to a group of healthy control.

**Methods:**

A total of 646 reproductive-aged women were randomly selected using the stratified, multistage probability cluster sampling method. The subjects were divided into five phenotypes: A (oligo/anovulation + hyperandrogenism + polycystic ovaries), B (oligo/anovulation + hyperandrogenism), C (hyperandrogenism + polycystic ovaries) and D (oligo/anovulation + polycystic ovaries). Hormonal and metabolic profiles and the prevalence of metabolic syndrome among these groups were compared using ANCOVA adjusted for age and body mass index.

**Results:**

Among women with PCOS (n = 85), those of groups A and C had higher serum levels of insulin and homeostatic model assessment for insulin%20resistance (HOMA-IR), compared to PCOS women of group D. Serum concentrations of cholesterol, low density lipoprotein, triglycerides and glucose in group A were higher than in other phenotypes, whereas the metabolic syndrome was more prevalent among group B.

**Conclusions:**

Women who had all three components of the syndrome showed the highest level of metabolic disturbances indicating that metabolic screening of the severest phenotype of PCOS may be necessary.

**Electronic supplementary material:**

The online version of this article (doi:10.1186/1477-7827-12-89) contains supplementary material, which is available to authorized users.

## Background

Polycystic ovary syndrome (PCOS) is one of the most common, complex and heterogeneous endocrine disorders [1–4] affecting 6-10% of women of fertile age [5, 6]. Although the etiology of PCOS is still unclear, genetic and environmental factors have been considered as possible contributors [1, 3, 7]. PCOS is complicated mainly with chronic anovulation (AnOvu), hyperandogenism (HA) and polycystic ovary manifestation (PCO) on ultrasound examination [2, 3, 8, 9]; it is also associated with metabolic disorders such as obesity, dyslipidemia, inflammation laboratory findings, high blood pressure, insulin resistance (IR) and metabolic syndrome (MetS) [2, 3, 7, 10], all of which lead to cardiovascular diseases [11–13].

IR is strongly linked to PCOS [10] and hypernandrogenism [14] and along with other hormonal irregularities has been reported to be higher among obese PCOS women than non-obese ones [1, 3, 15–17]; however its cause and effect relationship has not been clarified [18]. In contrast, IR is usually associated with MetS, although this higher prevalence of MetS has not always been reported in all studies conducted in PCOS women [16]. It seems that beside geographical and ethnical/racial variations [7, 16, 17], the association between PCOS and MetS is highly dependent on cut-offs defined for each of MetS components [19], PCOS criteria, type of study and PCOS phenotypes [18]. There are a limited number of studies on the metabolic aspects of various PCOS phenotypes including groups A (AnOvu + HA + PCO), B (AnOvu + HA), C (HA + PCO) and D (AnOvu + PCO) [3, 18]. It is still unclear that whether milder phenotypes have the same metabolic and reproductive consequences as more severe ones and whether we need to consider all of these metabolic complications and use strict screening for those with milder pictures [3, 18, 20]; some studies claim that phenotype A exhibits the severest endocrine and metabolic abnormalities [3, 20, 21], while phenotype D (the normoandrogenic phenotype) shows less [7]. Contrary to these, one study reported higher serum concentrations of insulin in group B and an insignificant upward trend of homeostatic model assessment of insulin%20resistance (HOMA-IR) among groups B and D [2], a difference which however disappeared after matching for age and body mass index (BMI) [22]; hence it seems that higher insulin and HOMA-IR could be related to a higher prevalence of obesity in these PCOS women, rather than its phenotypes per se [20, 21, 23].

Due to a lack of adequate population based studies on the metabolic aspects of the various PCOS phenotypes, we aimed to compare the metabolic parameters of four PCOS phenotypes to a group of non-PCOS controls in a community based study, conducted in the southwest of Iran.

## Methods

### Ethical approval

Ethical review board of the Research Institute for Endocrine Sciences see Additional file 1 approved the study proposal (initiation date of the study: 23/9/2010 and termination date of the study: 10/3/2012 ) and informed consent was obtained from all subjects see Additional file 2.

A stratified, multistage cluster with a probability in proportion to size procedure was used for the sampling method. The study design, recruitment process and data collection have been described previously in detail elsewhere [24]. In brief, a total of 646 women, aged 18-45 years from urban areas of three cities of Khouzestan province, including Ahvaz, Behbahan and Abadan, were randomly selected. Menopausal women, those who had undergone hysterectomy or bilateral oophorectomy and pregnant ones were excluded (overall n = 21). To minimize the effect of treatment bias, all other women, regardless of hormonal usage such as insulin sensitizers and oral contraceptive pills, were not excluded, but their hormonal and biochemical parameters were not statistically analyzed.

A standard questionnaire was completed for eligible women (n = 625). All participants underwent clinical examinations by trained staff of local medical universities/schools and their body weight, height, waist (WC), hip circumferences (HC) and blood pressure were measured and documented. BMI was calculated as weight in kilograms divided by height in meters squared (kg/m2). Acne was scored based on its number, type and distribution [25]. An overnight fasting venous blood sample was obtained from each subject on the second or third day of their spontaneous or progesterone induced menstrual cycles. All sera were stored at -80°C until the time of measurements. All study subjects were invited for transvaginal or transabdominal ultrasound scans of the ovaries, performed using either the 3.5-MHz transabdominal or 5-MHz transvaginal transducer by an experienced sonographer in each province. Ultrasound was performed as the same day as the blood samples were collected.

Insulin (Ins) was measured by immunoenzymometric assay (IEMA), (Mercodia, Uppsala, Sweden). Glucose (Glu), Triglycerides (TG), Total Cholesterol (TC), Low Density Lipoprotein (LDL) and High Density Lipoprotein (HDL) were measured by enzymatic colorimetery, (Pars Amazon Co. Tehran, Iran). HOMA-IR was calculated as fasting insulin (mIU/L) * fasting glucose (mg/dl) /405.

The intra- and inter-assay coefficients of variation for Ins were 0.9% and 1.1%, respectively; for Glu they were 1.3% and 2.9%, for TG 1.8% and 2.7%, for TC 0.8% and 2.8%, and for LDL 0.7% and 2.9%; and for HDL, these values were 0.9% and 3.3%, respectively.

We defined PCOS in our study using the Rotterdam (Rott) criteria, by which PCOS was defined as presence of two or more of the following: oligo/amenorrhea, clinical and/or biochemical hyperandrogenism, and polycystic ovaries [26]. Women were subdivided into 5 groups according to their manifestations: A) PCOS women who had oligo/anovulation, hyperandrogenism and polycystic ovaries, B) PCOS women with oligo/anovulation and hyperandrogenism, C) PCOS women with hyperandrogenism and polycystic ovaries, D) PCOS women with oligo/anovulation and polycystic ovaries, and E) non-PCOS women.

Hyperandrogenism (HA) was considered as clinical (the presence of hirsutism scores ≥ 8, using the modified Ferriman-Gallwey scores/acne/androgenic alopecia [24, 25, 27]) and/biochemical (circulating TT, FT, A4 and DHEAS levels > the 95th percentile for the studied women, who neither had clinical evidence of hyperandrogenism or menstrual disturbances, nor were they taking any hormonal medication) hyperandrogenism. Specifically, the upper normal limits were total T =0.89 ng/ml, A4 = 2.9 ng/ml, DHEAS =179 μg/dL and FAI =5.39. Oligo/anovulation (AnOvu) was defined when there was amenorrhea, or menstrual cycles longer than 35 days or less than 26 days [28]. Polycystic ovaries (PCO) were identified on ultrasonography if 12 or more follicles with a 2–9 mm diameter and/or increased ovarian volume were seen (10 cm3) [24, 29, 30].

Metabolic syndrome was defined by the clinical diagnostic criteria used for Iranian adult metabolic syndrome as the presence of any three of five of the following characteristics: (1) Waist circumference ≥ 95 cm; (2) Fasting TG ≥ 150 mg/dl or drug treatment for elevated triglycerides; (3) HDL cholesterol < 50 mg/dl or drug treatment for reduced HDL-C; (4) Systolic blood pressure ≥130 or diastolic blood pressure ≥ 85 mmHg or treatment with antihypertensive medication, and (5) Fasting glucose ≥ 100 mg/dl or treatment with diabetes medication [31].

### Statistical analysis

Continuous variables are presented as mean and standard deviation or median and the 25^th^ and 75^th^ percentages, following testing for normality, and categorical variables, are expressed as percentages. Demographic and anthropometric features between phenotypes are compared using one way ANOVA and/or the Kruskal-Wallis Hand or Pearson’s *χ*2 test, as appropriate. The association between PCOS manifestations with hormonal and metabolic parameters are analyzed using ANCOVA, adjusted for age and BMI. The association between MetS and phenotypical groups adjusted for age and BMI are analyzed using logistic regression model. Data analysis was performed using the SPSS 15.0 PC package(SPSS Inc., Chicago, IL) and statistical significance was set at P < 0.05.

## Results

Of the 602 participants who completed the study, 85 women met the PCOS criteria; the most common phenotype among them was C (49.4%) i.e. they met two criteria of PCOS including hyperandrogenism and polycystic ovaries; group B (22.4%), D (15.3%), and A (12.9%) followed in that order. Non-PCOS women were significantly older than those with PCOS (33.9 ± 7.6 years vs 29.07 ± 7.7 years; P < 0.001); however there were no significant differences in mean weight, height, BMI, WC, HC, waist to hip ratio (WHR) between PCOS and non-PCOS participants. Table 1 shows the participants’ features stratified according to various PCOS phenotypes and their non-PCOS counterparts.

**Table 1:** **Features of the participants, classified to 4 phenotypical groups and non-PCOS women**

Phenotype	A (n = 11)	B (n = 19)	C (n = 42)	D (n = 13)	Normal (n = 517)
Variables	AnOvu + HA + PCO	AnOvu + HA	HA + PCO	AnOvu + PCO	
**Age (years)**	25.6 ± 7.0	31.1 ± 7.8	30.3 ± 7.5	24.7 ± 6.8	33.9 ± 7.6
**≤25**	6 (54.5%)	7 (36.8%)	13 (31%)	8 (61.5%)	93(18%)
**26-35**	4 (36.4%)	5 (26.3%)	19 (45.2%)	4 (30.8%)	186(36%)
**36-40**	0	5 (26.3%)	4 (9.5%))	0	106(20.5%)
**≥41**	1 (9.1%)	2 (10.5%)	6 (14.3%)	1 (7.7%)	132(25.5%)
**Weight (Kg)**	64.1 ± 11.9	66.2 ± 13.0	68.8 ± 11.3	57.7 ± 5.9	67.8 ± 13.1
**Height (cm)**	159.1 ± 4.4	158.2 ± 6.1	158.9 ± 5.2	156.3 ± 11.7	159.5 ± 6.2
**BMI (Kg/m** ^**2**^ **)**	25.4 ± 5.0	26.4 ± 4.8	27.2 ± 4.4	24.1 ± 5.5	26.6 ± 5.0
**Waist (cm)**	76.1 ± 11.7	83.3 ± 11.6	81.8 ± 8.9	74.5 ± 6.2	81.5 ± 10.7
**Hip (cm)**	94.5 ± 11.6	98.8 ± 11.9	100.48 ± 12.6	92.5 ± 6.5	100.4 ± 12.0
**WHR (cm)**	0.80 ± 0.07	0.85 ± 0.05	0.81 ± 0.10	0.82 ± 0.06	0.81 ± 0.09
**Systolic Blood Pressure (mmHg)**	105.6 ± 12.5	106.4 ± 11.8	109.0 ± 11.1	107.9 ± †8.9	109.6 ± 12.9
**Diastolic Blood Pressure (mmHg)**	65.0 ± 9.7	67.0 ± 10.4	70.4 ± 9.6	66.2 ± 9.3	68.9 ± 10.4
**Hirsutism**	8 (72.7%)	12 (63.1%)	33 (78.5%)	0†॥‡	129(25%)
**Acne**	4 (36.4%)	7 (36.8%)	18 (42.9%)	4 (30.8%)	78(15.1%)

versus Normal, P<0.05.

† versus group A, P<0.05.

॥ versus group B, P<0.05.

‡ versus group C, P<0.05.

**Figure 1:**
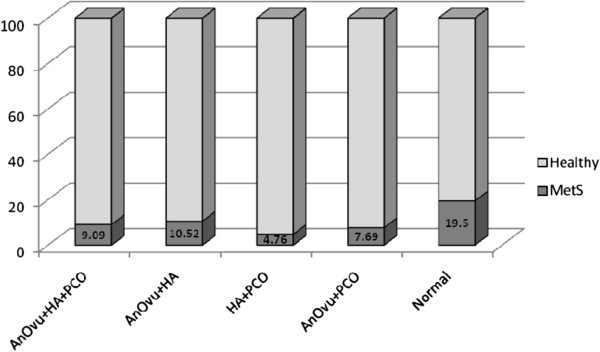
**Prevalence of MetS among participants, classified to 4 phenotypical groups and in non –PCOS women.**

PCOS women showed significantly higher serum levels of total testosterone (TT) (0.28 ± 0.28 nmol/l; P < 0.001), androstenedione (A4) (2.51 ± 1.62 ng/ml; P < 0.001), dehydroepiandrosterone sulfate (DHEAS) (166.49 ± 83.28 μg/dl; P < 0.001), free androgen index (FAI) (2.32 ± 2.23; P < 0.001) and prolactin (PRL) (22.30 ± 14.43 ng/ml; P = 0.002) versus non-PCOS ones. Table 2 shows hormonal and metabolic profiles of the 4 PCOS phenotypes vs the non-PCOS ones. The prevalence of MetS among PCOS women was 7.1%, and19.53% in non-PCOS women. The prevalence of MetS among 4 PCOS phenotypes and non-PCOS ones is presented in Figure 1.

**Table 2:** **The serum levels of the hormones and metabolic profile, classified to 4 phenotypical groups and in non-PCOS women**

Phenotype	A (n = 11)	B (n = 19)	C (n = 42)	D (n = 13)	E (n = 517)
Variables	AnOvu + HA + PCO	AnOvu + HA	HA + PCO	AnOvu + PCO	Normal
**Testosterone (nmol/l)**	0.4 ± 0.2	0.3 ± 0.4	0.2 ± 0.2	0.1 ± 0.1^†^	0.1 ± 0.2
**Androstendione (ng/ml)**	3.7 ± 1.9	3.2 ± 2.1 ^‡^	2.0 ± 1.1 ^†^	1.7 ± 0.7^†‡^	1.4 ± 0.9
**DHEAS (μg/dl)**	205.7 ± 79.6	188.7 ± 93.4 ^‡^	161.2 ± 81.5	116.7 ± 52.1^†‡^	119.7 ± 73.3
**SHBG (nmol/l)**	50.1 ± 39.62	49.7 ± 31.5	44.6 ± 30.2	54.4 ± 29.3	54.8 ± 34.9
**FAI**	2.8 ± 1.7	2.8 ± 2.6	2.3 ± 2.3	1.0 ± 1.0	1.2 ± 1.5
**LH (μU/ml)**	7.5 ± 5.9	7.8 ± 7.7	6.7 ± 5.4	6.0 ± 4.6	6.3 ± 5.9
**FSH (μU/ml)**	7.5 ± 1.17	8.4 ± 3.5	10.8 ± 12.0	8.3 ± 1.8	11.6 ± 10.0
**Prolactin (ng/ml)**	22.8 ± 12.0	19.9 ± 16.3	22.8 ± 15.3	23.4 ± 10.7	19.6 ± 20.0
**TSH (μU/ml)**	2.4 ± 1.3	2.4 ± 1.5	3.2 ± 2.7	1.8 ± 0.6	2.8 ± 2.7
**Free T4 (picomol/l**	15.7 ± 1.9	16.0 ± 3.4	15.7 ± 2.0	17.0 ± 2.6	15.5 ± 2.2
**Insulin (μU/ml)**	13.1 ± 10.6	7.7 ± 2.8	10.5 ± 13.0	5.7 ± 3.8 ^†‡^	9.7 ± 9.4
**HOMA-IR**	3.1 ± 2.7	1.7 ± 0.7	2.1 ± 1.8	1.2 ± 0.9 ^†‡^	2.5 ± 3.0
**Cholesterol (mg/dl)**	167.5 ± 35.8	159.4 ± 24.2	165.4 ± 40.2	156.5 ± 34.4	169.5 ± 34.4
**HDL (mg/dl)**	47.2 ± 9.8	49.4 ± 13.3	49.1 ± 11.0	46.4 ± 12.0	46.4 ± 11.9
**LDL (mg/dl)**	97.0 ± 30.5	89.2 ± 24.1	96.9 ± 39.2	93.1 ± 29.0	98.8 ± 29.5
**Triglycerides (mg/dl)**	116.3 ± 57.3	103.5 ± 49.5	98.0 ± 38.6	83.9 ± 40.1	122.6 ± 80.5
**Glucose (mg/dl)**	95.5 ± 7.8	94.1 ± 22.0	90.3 ± 11.8	87.5 ± 7.6	100.3 ± 48.0

All analyses by ANCOVA adjusted for age and BMI.

versus group E, P < 0.05.

^†^versus group A, P < 0.05.

^‡^versus group C, P < 0.05.

Pearson’s *χ*2 test revealed that non-PCOS participants indicated a significantly higher number of women with MetS, compared to PCOS ones (P = 0.005). Regarding the phenotype groups, Pearson’s *χ*2 test showed that group C included fewer number of women with MetS compared to those without PCOS (P = 0.01). Multivariate logistic regression showed that after adjustment for age and BMI, the prevalence of MetS in group C was significantly lower than in the non-PCOS women (OR = 0.22, 95% CI: 0.05, 0.99; P = 0.04).

## Discussion

The present community-based study revealed that PCOS phenotypes with hyperandrogenism (A, B and C) had the worst metabolic presentations in terms of insulin resistance and metabolic syndrome. Furthermore the lipid and glucose profiles of women with mild phenotype (D) were better than the other PCOS groups, even non-PCOS ones. Our findings also indicate that concurrency of the three PCOS symptoms leads to increased severity of metabolic disorders, especially hyperinsulinemia which may be related to more severe hyperanrogenemia.

Similar to our findings, a study conducted on 93 Polish women with PCOS, showed elevated serum concentrations of TT, Chol and LDL in the classic phenotype of PCOS i.e. group A [2]; other researchers have also reported the worst disturbances [3, 32]. In a study from Greece, IR was higher among groups A and B, but had the lowest levels among group C (HA + PCO), it was concluded that group C had the lowest risk of cardiovascular diseases [20].

It is noteworthy that although PCOS women have shown higher risk of cardiovascular diseases, yet hyperandrogenism has not been proven to be the main contributor to future cardiovascular diseases [6]; however PCOS has been diagnosed in 55-80% of hyperandrogenic patients [1]. As hyperinsulinemia can increase androgen secretion, a PCOS characteristic, it is believed that hyperinsulinism might contribute to PCOS development [32, 33], and can also lead to the development of Glu intolerance and type 2 diabetes [3, 4]. necessitating more attention be given to ethnic differences in insulin receptor action in PCOS [7].

Our findings of lower lipid- and Glu- profile-related disorders in normoandrogenic patients (group D) were consistent with the androgen excess and the PCOS society’s claim that PCOS should initially be considered an androgen excess disorder [20]. Group D without HA symptoms is reported to have fewer metabolic abnormalities, lower BMI and WC [7] findings in agreement with ours; in contrast a study done in Italy on 220 PCOS women and 144 age and BMI matched controls, found no association between IR and HA compared to non-PCOS women [33]. Lower Glu in PCOS women may reflect the increased glycolysis in muscles and decreased gluconeogenesis in the liver as a result of PCOS pathology [3].

Two studies documented an association between AnOvu and metabolic disorders [10, 32], although another reported that the serum levels of TG and TC remain in the normal range among PCOS women [6]. In the Rizzo et al study (2009), the ovulatory PCOS phenotype showed lower TC, TG, LDL and higher HDL levels, compared to anovulatory ones [6]; findings similar to ours of an insignificant lower prevalence of MetS in group C (ovulatory group); since in our study more PCOS patients belonged to phenotype C, this is a favorable finding. Phenotype B (AnOvu + HA) showed the highest prevalence of MetS among 4 PCOS groups; they were older and also had higher WHR, indicating higher incidence of androgenic obesity. The higher prevalence of MetS among non-PCOS women is probably due to older age and higher BMI, neither remaining significant after adjustment for these two variables. These differences between groups is not be related to the selection bias as aging decreases the prevalence of PCOS symptoms [12]; PCOS patients are hence more likely to be younger than non-PCOS ones.

The slight differences between results of studies are probably due to genetic and environmental factors, in addition to heterogeneity of PCOS phenotypes. Similar to our findings, in another study conducted in Iran, the prevalence of MetS in phenotype B was reported the highest. A study conducted by Mehrabian et al has shown that PCO has negative association with MetS [16] and the study of Amato et al from Italy also showed that MetS is the least prevalent in group C [33].

This study does have its strengths, such as using a community-based sample instead of recruitment from a referral centre. In addition, we used national cut points of WC and HOMA-IR for the definition of abdominal obesity and IR, respectively. To mention the limitations, since aging decreases serum androgen levels and increases the prevalence of IR and MetS [20], a limitation of our study was that women of non-PCOS group were significantly older than PCOS ones; however we tried to eliminate this effect by adjusting for age in the models. Furthermore the age difference between our 4 phenotypical groups was not significant; therefore our comparative analysis may not have been influenced by the age variation. Another potential limitation that needs to be mentioned is that we used HOMA-IR as a surrogate marker for assessing of IR; in spite of a good correlation between HOMA-IR and gold standard clamp methods [34, 35] the assessment might be inaccurate.

## Conclusions

In conclusion, our results indicate that, women with hyperandrogenism exhibit the worst metabolic features. As a result screening of phenotypes with hyperandrogenic symptoms for detection of metabolic abnormalities is highly recommended in the scope of PCOS. Further longitudinal population based studies, with a prospective risk assessment approach in larger sample sizes are needed to analyze the long-term effects of metabolic profiles of each phenotype.

## Electronic supplementary material

Additional file 1: **Ethical approval.** (DOCX 973 KB)

Additional file 2: **Consent.** (DOCX 16 KB)

## Abbreviations

A4: Androstenedione
AnOvu: Anovulation
BMI: Body mass index
DHEAS: Deheydroepiandroston
FAI: Free androgen index
Glu: Glucose
HA: Hyperandrogenism
HC: Hip circumference
HDL: High density lipoprotein
IEMA: Immunoenzymometric assay
Ins: Insulin
IR: Insulin resistance
LDL: Low density lipoprotein
MetS: Metabolic syndrome
PCO: Polycystic ovary (seen by sonography)
PCOS: Polycystic ovary syndrome
PRL: Prolactin
TC: Total cholesterol
TG: Triglycerides
TT: Total testosterone
WC: Waist circumference.
