# Supplementary material for: The prevalence of metabolic disorders in various phenotypes of polycystic ovary syndrome: a community based study in Southwest of Iran
Source: Reprod Biol Endocrinol. 2014 Sep 16;12:89. doi: 10.1186/1477-7827-12-89 (PMC4180586; doi:10.1186/1477-7827-12-89)
Supplement: Supplementary file 1 — Additional file 1: Ethical approval. (DOCX 973 KB) [file 12958_2014_1262_MOESM1_ESM.docx]

**
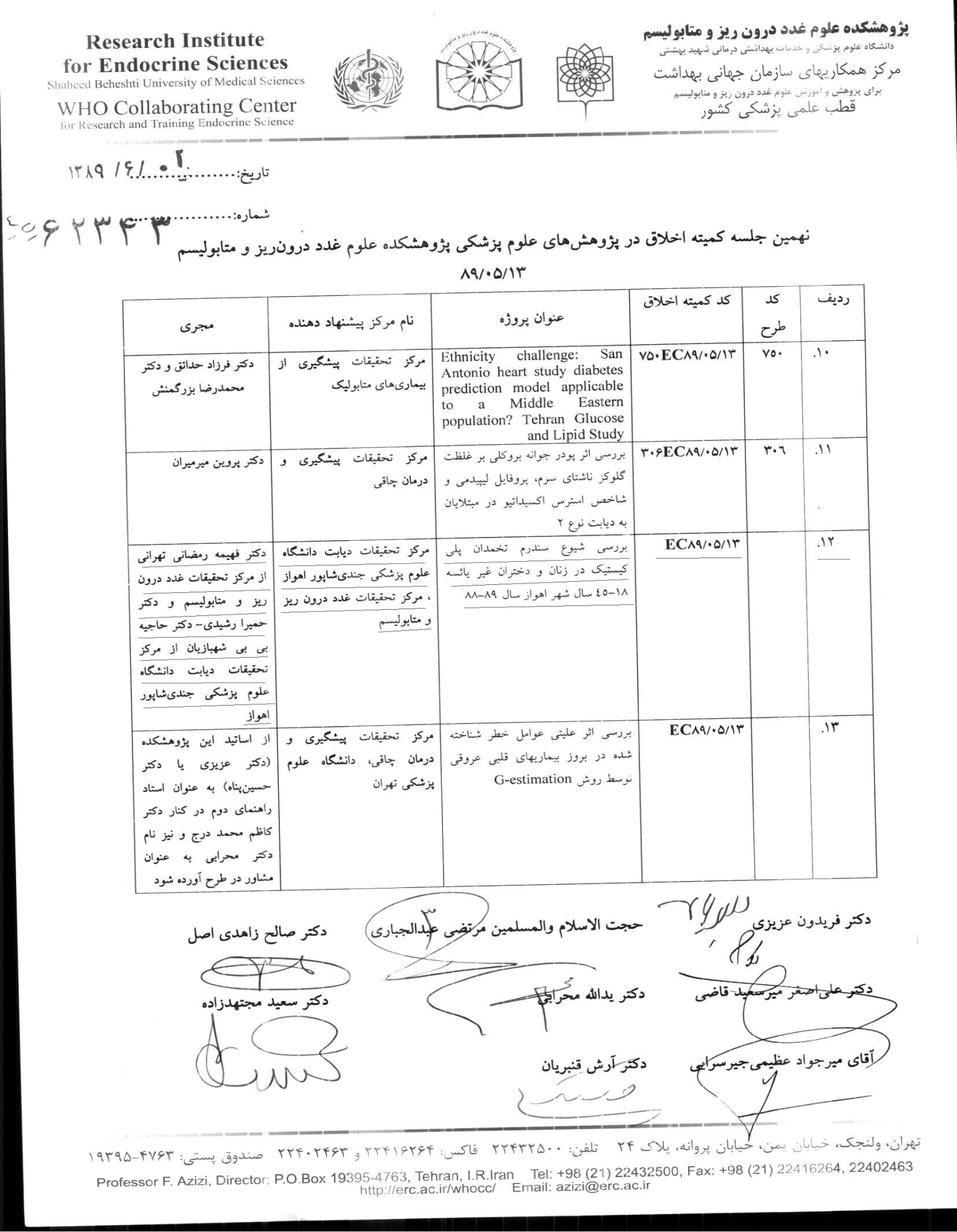
Research institute for endocrine sciences**

Shaheed Beheshti University of Medical Sciences

WHO Collaborating Center

for research and training endocrine sciences

Date: 2010/8/24

Number: 62343

**The ninth meeting on ethics in medical sciences in endocrine and metabolism research center
Held on: 2010/8/4**

| Row | Code | Ethical Committee Code | Title | Research Center/s | Project Designers |
| --- | --- | --- | --- | --- | --- |
| 12 |  | EC89/05/13 | the prevalence of polycystic ovary syndrome among premenopausal women (15-49 years old) from Southwest of Iran, 2009-2010 | Diabetes Research Center, Ahvaz Jundishapur University of Medical Sciences & Research institute for endocrine sciences,Shaheed Beheshti University of Medical Sciences | Dr. Fahimeh Ramezani Tehrani, Dr. Homeira Rashidi and Dr. Hajieh Bibi Shahbazian |
