# Supplementary material for: The prevalence of metabolic disorders in various phenotypes of polycystic ovary syndrome: a community based study in Southwest of Iran
Source: Reprod Biol Endocrinol. 2014 Sep 16;12:89. doi: 10.1186/1477-7827-12-89 (PMC4180586; doi:10.1186/1477-7827-12-89)
Supplement: Supplementary file 2 — Additional file 2: Consent. (DOCX 16 KB) [file 12958_2014_1262_MOESM2_ESM.docx]

| فرم رضايت نامه شركت كنندگان در طرح تحقيقاتي تعیین میزان شیوع سندرم تخمدان پلی کیستیک در میان دختران و زنان غیر یائسه 18-45 ساله جنوب غربی ایران |
| --- |

## هموطن گرامي : کرج □ تهران □ شماره فرد □ □ □ □

## سلامت خانواده حاصل سلامت تك تك اعضاي آن است . در اين ميان سلامت زنان به عنوان ركن اصلي خانواده بسيار حائز اهميت است. بسياري از بيماريهايي كه سلامت زنان و دختران را تهديد مي كند در مراحل اوليه فاقد علامت است و در صورت عدم تشخيص به موقع و درمان مناسب مي تواند عواقب جبران ناپذيري را در سلامت خانواده داشته باشد. به منظور تعيين شيوع و علائم بيماريهايي كه سلامت باروري زنان را تهديد مي كند اين تحقیق توسط پژوهشكده علوم غدد درون ريز و متابوليسم دانشگاه علوم پزشكي و خدمات بهداشتي درماني شهيد بهشتي طراحی و اجرامي گردد . شما به شكل كاملا تصادفي جهت معاينه و انجام آزمايشات تخصصي انتخاب شده اید تا با كمك اطلاعات بدست آمده بتوان براي سلامت جامعه زنان كشور برنامه ريزي و اقدام كرد.

## بديهي است تمامي معاينات عمومي، تخصصي، آزمايشگاهي در اين طرح كاملا رايگان انجام خواهد شد و در صورت نياز به درمان، در اين زمينه راهنمايي يا مشاوره لازم ارائه خواهد شد. اطلاعات گردآوري شده محرمانه بوده و به منظور مشخص نبودن نام شركت كننده از كد اختصاصي استفاده خواهد شد و اصل رازداري همواره لحاظ خواهد گرديد. شركت شما در اين معاينات كاملا اختياري است و چنانچه در هر مرحله تمايل به پاسخگويي به سوالات نداشته يا راضي به انجام معاينات نباشيد مي توانيد انصراف خود را اعلام نماييد.

## اينجانب------------------- با اطلاع از مطالب گفته شده بالا، با آگاهي و رضايت كامل با امضاي اين فرم در اين بررسي شركت مي كنم .

## نام و نام خانوادگي امضاء

## Written informed consent of participants in the research project entitled to determine the prevalence of polycystic ovary syndrome among premenopausal women (18-45 years old) from Southwest of Iran

Karaj Tehran**** code 

Dear Compatriot,

Family health is achieved through the health of each family member. Meanwhile, women’s health, as the main pillars of the family is very important. Many diseases that threaten the health of women and girls are asymptomatic in early stages and in the absence of early diagnosis and appropriate treatment may lead to consequences on the health of the family. This study has been designed by research institute of Endocrinology and Metabolism, Shahid Beheshti University of Medical Sciences, and aims to determine the prevalence of the diseases and their symptoms which threaten women’s reproductive health. You are randomly selected for doing examinations and laboratory tests in order to plan for the health of women in the society through obtained data.

NOTE: All general and special examinations and laboratory tests in this project will be done completely free, and if necessary, any needed advice or treatment in this field will be provided. Collecting information will be confidential and in order not to specify participants’ names, specific codes will be used and secrecy always will be considered. Your participation is completely optional and if you do not incline to respond to the questionnaire or do any of examinations, you can do cancel your participation in this study.

I, ------------------- am aware of all written or verbal mentioned points, and sign this consent to participate in this study.

NAME: Signature
